# Survey of selected viral agents (herpesvirus, adenovirus and hepatitis E virus) in liver and lung samples of cetaceans, Brazil

**DOI:** 10.1038/s41598-023-45315-9

**Published:** 2024-02-01

**Authors:** C. Sacristán, A. C. Ewbank, A. Duarte-Benvenuto, I. Sacristán, R. Zamana-Ramblas, S. Costa-Silva, V. Lanes Ribeiro, C. P. Bertozzi, R. del Rio do Valle, P. V. Castilho, A. C. Colosio, M. C. C. Marcondes, J. Lailson-Brito, A. de Freitas Azevedo, V. L. Carvalho, C. F. Pessi, M. Cremer, F. Esperón, J. L. Catão-Dias

**Affiliations:** 1 Centro de Investigación en Sanidad Animal (CISA-INIA), CSIC, Carretera Algete-El Casar de Talamanca, Km. 8,1, 28130 Valdeolmos, Madrid, Spain; 2 School of Veterinary Medicine and Animal Sciences, University of São Paulo, São Paulo, SP Brazil; 3 Instituto Biopesca, Praia Grande, SP Brazil; 4 São Paulo State University - UNESP, São Vicente, SP Brazil; 5 Instituto Ecoema de Estudo e Conservação do Meio Ambiente, Peruíbe, SP Brasil; 6 Universidade do Estado de Santa Catarina-UDESC, Laguna, SC Brazil; 7 Instituto Baleia Jubarte, Caravelas, BA Brazil; 8 Laboratório de Mamíferos Aquáticos e Bioindicadores ‘Profa Izabel M. G. do N. Gurgel’ (MAQUA), Faculdade de Oceanografia, Universidade do Estado do Rio de Janeiro, Rio de Janeiro, RJ Brazil; 9 Associação de Pesquisa e Preservação de Ecossistemas Aquáticos, Caucaia, CE Brazil; 10 Instituto de Pesquisas Cananéia (IpeC), Cananéia, SP Brazil; 11 Laboratório de Ecologia e Conservação de Tetrápodes Marinhos e Costeiros – TETRAMAR, Universidade da Região de Joinville – UNIVILLE, São Francisco Do Sul, SC Brazil; 12 Universidad Europea, Villaviciosa de Odon, Spain

**Keywords:** Viral infection, Conservation biology, Infectious diseases

## Abstract

Hepatic and pulmonary lesions are common in cetaceans, despite their poorly understood viral etiology. Herpesviruses (HV), adenoviruses (AdV) and hepatitis E virus (HEV) are emerging agents in cetaceans, associated with liver and/or pulmonary damage in mammals. We isolated and molecularly tested DNA for HV and AdV (n = 218 individuals; 187 liver and 108 lung samples) and RNA for HEV (n = 147 animals; 147 liver samples) from six cetacean families. All animals stranded or were bycaught in Brazil between 2001 and 2021. Positive-animals were analyzed by histopathology. Statistical analyses assessed if the prevalence of viral infection could be associated with the variables: species, family, habitat, region, sex, and age group. All samples were negative for AdV and HEV. Overall, 8.7% (19/218) of the cetaceans were HV-positive (4.8% [9/187] liver and 11.1% [12/108] lung), without HV-associated lesions. HV-prevalence was statistically significant higher in Pontoporiidae (19.2%, 10/52) when compared to Delphinidae (4.1%, 5/121), and in southeastern (17.1%, 13/76)—the most industrialized Brazilian region—when compared to the northeastern region (2.4%, 3/126). This study broadens the herpesvirus host range in cetaceans, including its description in pygmy sperm whales (*Kogia breviceps*) and humpback whales (*Megaptera novaeangliae*). Further studies must elucidate herpesvirus drivers in cetaceans.

## Introduction

Cetaceans are considered of special interest due to their fundamental role in the functionality of aquatic ecosystems, classified as “sentinel organisms”^1,2^. Additionally, cetaceans are especially important in terms of public health because they share the coastal ecosystems and feed on species similar to those present in human diets^2^.

Cetacean populations may be negatively affected by direct and indirect impacts of anthropogenic origin, such as fishing activities, boat collision, and chemical, noise or biological pollution^3,4^. Such factors are especially relevant to coastal marine species, more frequently exposed to pollution by certain organic contaminants (e.g., polychlorinated biphenyls) and pesticides of agricultural use, eutrophication processes caused by excessive organic material from agricultural, livestock and urban origin, as well as pathogens of terrestrial source^5,6^. In cetaceans, emerging infectious diseases are probably among the main long-term consequences of the negative impact of human activities^2,7^. Among the diseases that affect cetaceans, hepatic and pulmonary ailments are classified as relevant and relatively common^8–10^. Hepatic lesions are mainly characterized by decreased toxin metabolism and subsequent elevation on the risk of immunosuppression and associated diseases, and reproductive impairment^11^. Pulmonary lesions can cause dyspnea and buoyancy problems^12^, compromising the immersion capacity of cetaceans^13^.

Regarding hepatic lesions, parasitic diseases are one of the most frequently reported infectious causes in cetaceans, with special mention to trematodes (e.g. *Campula* spp.), followed by Apicomplexa protozoans (*Sarcocystis* spp. and *Toxoplasma gondii*)^14–16^. Among the studies on viral hepatic diseases, one must highlight the detection of hepatitis E virus (HEV), capable of causing hepatitis in common bottlenose dolphins (*Tursiops truncatus*), and experimental hepatitis A virus infection in dolphin cell line^17,18^. Some other viral description in liver samples include a gammacoronavirus identified in a beluga whale (*Delphinapterus leucas*) with acute necrotic hepatitis^19^, and systemic morbillivirus infections in cetaceans with periportal lymphoplasmacytic hepatitis^20^. Finally, particles similar to herpesvirus were detected in an Indo-Pacific finless porpoise (*Neophocaena phocaenoides*) with necrotizing hepatitis^21^. Additionally, adenoviruses—agents related to hepatitis in other taxonomic groups, have also been described in cetaceans^22–24^.

One of the most important respiratory findings in cetaceans is pneumonia^25^. Among its etiologies, bacterial pneumonia is a common cause of death in cetaceans^9,10^. Other infectious etiologies of respiratory disease are mycotic (e.g., *Aspergillus* spp.) and parasitic infections (e.g., *Toxoplasma gondii* and different nematodes and trematodes), with the latter considered major causes of respiratory illness^26–28^. Viruses are also recognized as primary causes of respiratory disease in cetaceans, as observed in pneumonia cases caused by morbillivirus infection^29^. Moreover, a novel polyomavirus has been associated with thracheobronchitis in a common dolphin (*Delphinus delphis*)^30^, and alphaherpesviruses were linked to interstitial pneumonia in common bottlenose dolphins^31^. Additionally, alpha- and gammaherpesviruses have been molecularly detected in lung samples of animals with respiratory disease, even though an etiological relationship has not been established yet^32–35^. Other viruses (e.g., Avian influenza virus, coronavirus, parainfluenza virus, and adenovirus) able to cause respiratory disease in mammals have also been described in cetaceans, although without associated respiratory lesions^19,22–24,36^. Of note, herpesviruses and adenoviruses are recognized as significant causes of interstitial pneumonia in other mammal taxa^37,38^.

Despite the description of some infectious agents in cetacean liver and lung samples, the etiology of several of these hepatic and pulmonary lesions has not been established^39–41^. Additionally, there is also little knowledge about the potential role of herpesviruses and adenoviruses as causes of hepatic and pulmonary lesions in cetaceans, and the correlation of HEV with hepatitis in this taxon.

The prevalence of pathogens can be modified by habitat degradation, usually linked to anthropization^42,43^. The main goals of this study were to: (i) compare the prevalence of selected infectious agents (herpesvirus, adenovirus and HEV) in coastal, pelagic, and mixed (both habitats) species and among regions of Brazil (northeastern, southeastern and south), to indirectly evaluate the impact of human activity on their health status; and (ii) determine if the selected agents are associated with hepatic or pulmonary lesions in cetaceans.

## Results

### Molecular analyses

#### Herpesvirus

Overall, the herpesvirus prevalence was 8.7% (19/218): 7 cetaceans were positive only in the liver, 10 only in the lung, and 2 in liver and lung samples (Table 2).

Regarding tissue type, 9 out of 187 (4.8%) cetacean liver samples were positive to herpesvirus by DPOL or gB PCR protocols, while 12 of 108 (11.1%) cetacean lung samples were herpesvirus-positive by the same techniques. Detailed information about the positive tissues per case is displayed in Table 2. Out of the herpesvirus-screened samples, 13 were positive to DPOL PCR (6 liver samples and 7 lung samples) and 11 to gB PCR (4 liver samples and 7 lung samples) (Table 2).

We detected herpesviruses in the families Pontoporiidae (19.2%, 10/52), Kogiidae (10.5%, 2/19), Balaenopteridae (5%, 1/20), Delphinidae (4.1%, 5/121), and Ziphiidae (1/2), while all Physeteridae were negative. According to the species, we found herpesviruses in franciscana (*Pontoporia blainvillei*, 19.2% [10/52]), Guiana dolphin (*Sotalia guianensis*, 5.1% [3/59]), Atlantic spotted dolphin (*Stenella frontalis*, 2/9), dwarf sperm whale (*Kogia sima*, 7.1% [1/14]), pygmy sperm whale (*Kogia breviceps*, 1/5), Gervais's beaked whale (*Mesoplodon europaeus*, 1/1), and humpback whale (*Megaptera novaeangliae*, 5.6% [1/18]) (Table 2). Regarding sex, we detected herpesvirus in 8.6% (8/93) females and 8.9% (11/124) males. In terms of age class, herpesvirus infection was identified in 10% (7/70) of the calves, 10.5% (6/57) of the juveniles, and 6.7% (6/89) of the adults, while the two analyzed fetuses were negative. Herpesviruses were detected in animals found in the southeastern (17.1%, 13/76), northeastern (2.4%, 3/126), and 18.8% (3/16) southern regions of Brazil, and in coastal (11.4%, 13/114), pelagic (4.5%, 3/67) and mixed habitat (8.1%, 3/37) species.

##### Alphaherpesvirus

Overall, 2.8% (6/218) of the cetaceans presented alphaherpesviruses in liver and/or lung samples. Aside from the alphaherpesvirus-positive dwarf sperm whale MM579 and the Guiana dolphin MM731 described in the study by Sacristán et al.^32^, we identified four novel cases positive to alphaherpesviruses. Specifically, another Guiana dolphin (MM672), an Atlantic spotted dolphin (ii230353), a humpback whale (Mn939), and a Gervais's beaked whale (MM382).

The Guiana dolphin MM672 presented a novel alphaherpesvirus DPOL sequence in liver, with a highest nucleotide (nt) similarity (98.4%) with sequences previously identified in a common dolphin (MG437205) in Portugal and two striped dolphins (*Stenella coeruleoalba*) of Italy and Spain (KX822073 and GQ888670, respectively). The deduced aa sequence had 100% identity to sequences described in the striped dolphins mentioned above.

The Atlantic spotted dolphin ii230353 had a DPOL sequence in lung, with the highest nt (98.4%) identity to alphaherpesviruses described in another Atlantic spotted dolphin of Brazil (MF999155), in two striped dolphins of the Mediterranean coast of Italy and Spain (KX822073, GQ888670) and in a common dolphin (MG437205) of Portugal. The deduced aa sequence was identical to those from the striped dolphins commented above (KX822073, GQ888670), and to an additional alphaherpesvirus found in a striped dolphin of the Canary Islands, Spain (KJ156332).

The humpback whale Mn939 presented a DPOL sequence in the lung, with the highest nt and aa identities (both of 95.3%) to an alphaherpesvirus (KP995686) found in a fin whale (*Balaenoptera physalus*) of the Spanish Mediterranean coast. The initial part of our sequence presented 15 bp that were not present in the fin whale sequence.

The Gervais's beaked MM382 whale had DPOL sequences in the lung, spleen and heart, identical among them, which presented the highest nt (99%) and aa (98.4%) similarities with an alphaherpesvirus sequence identified in a Gervais's beaked whale from the Canary Islands, Spain (MZ066758). Of note, our nt sequence was slightly longer than the one from the Canary Islands (208 vs. 194 bp).

##### Gammaherpesvirus

Overall, 6% (13/218) of the cases presented gammaherpesviruses in liver and/or lung samples, i.e., six franciscanas previously published by Exposto Novoselecki et al.^34^, another four novel franciscana cases (MM525, ii94246, ii126142, and ii160187), a pygmy sperm whale (ii179266), an Atlantic spotted dolphin (ii213578) and a Guiana dolphin (ii137962) (Table 2). Additionally, another Atlantic spotted dolphin (ii230353) presented a gammaherpesvirus, but only in kidney.

The DPOL and gB herpesvirus gammaherpesvirus sequences detected in the four novel franciscana cases were 100% identical to those previously identified by Exposto Novoselecki et al.^34^ in franciscanas from São Paulo state, Brazil. In our animals, 3 gB sequences were found in liver and 1 DPOL and 2 gB sequences were detected in lung. In cases ii126142 and ii160187, it was possible to analyze other tissues aside from liver, using DPOL and gB PCRs, with positives to DPOL in the spleen and mesenteric lymph node (ii160187), and to DPOL and gB in the duodenum and thymus (ii126142), presenting the same sequences described above (Table 2).

In the pygmy sperm whale (ii179266), a highly divergent DPOL sequence was found in liver, with greater nt similarity (61.4%) with a gammaherpesvirus sequence (KP721221) previously described in a red lynx (*Lynx rufus*) in the United States, and a greater aa similarity (60.0%) with a gammaherpesvirus amplified in an Asian elephant (*Elephas maximus*) kept under human care in Germany (JF705864).

In the lung of the Atlantic spotted dolphin ii213578, we identified a gammaherpesvirus gB sequence highly similar (96.2% nt and 100% aa identity) to a gammaherpesvirus sequence of a Bolivian river dolphin (*Inia boliviensis*) from Brazil (MZ209258). A gB sequence 100% identical to the one found in the lung of the Atlantic spotted dolphin ii213578 was detected in the kidney of the Atlantic spotted dolphin ii230353 (an animal coinfected with an alphaherpesvirus, as described above).

A gammaherpesvirus was identified in the lung of the Guiana dolphin ii137962, but it was not possible to obtain DPOL sequences of enough quality to perform identity analyses.

The five novel HV DNA polymerase nt sequences obtained from the positive Atlantic spotted dolphin (ii230353), Guiana dolphin (MM672), Gervais's beaked whale (MM382), humpback whale (Mn939) and pygmy sperm whale (ii179266) were submitted to GenBank under accession numbers OQ980334 to OQ980338, respectively. The two gB sequences retrieved in the Atlantic spotted dolphins ii213578 and 230,353 were submitted to GenBank under accession numbers OQ926585 and OQ926586, respectively.

#### Adenovirus and hepatitis E virus

None of the 187 cetacean liver and 108 lung samples tested for adenovirus were PCR-positive. All of the 147 RNA extractions of liver samples were negative to hepatitis E virus by RT-PCR.

### Phylogenetic results

**Figure 1:**
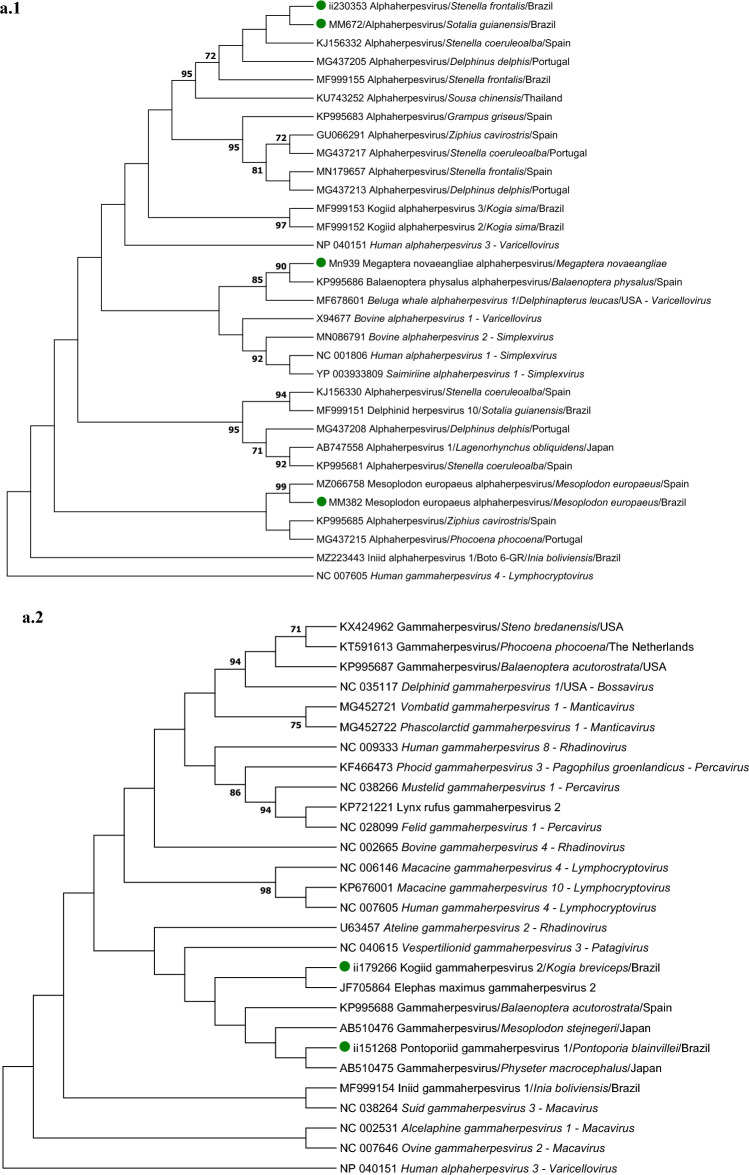

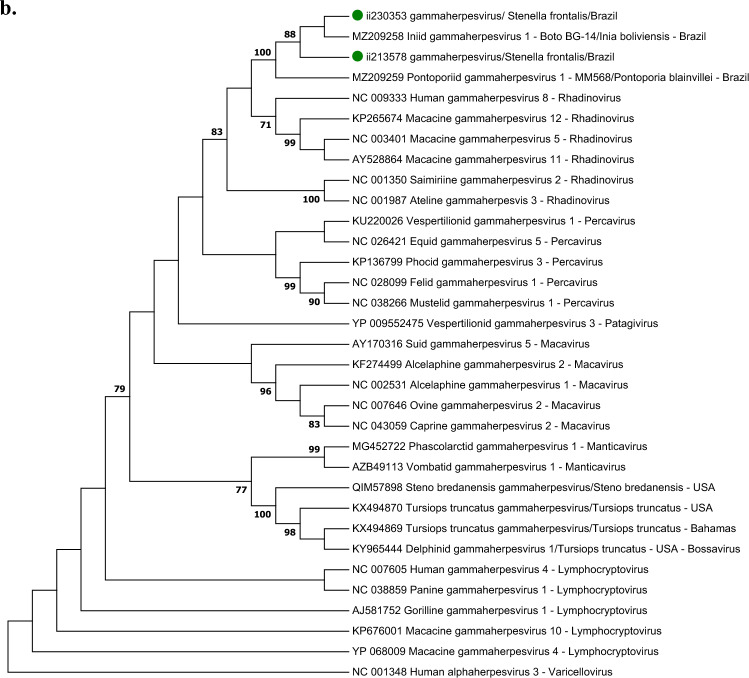
(**A**) Maximum likelihood phylogram of the herpesvirus DNA polymerase (DPOL) deduced amino acid sequences: (i) obtained in this study (green dots), (ii) the closest to these sequences from GenBank, (iii) alpha- and gammaherpesvirus sequences retrieved from other cetacean species, (iv) sequences of alpha- and gammaherpesvirus species recognized by the International Committee on Taxonomy of Viruses (ICTV); the DPOL alphaherpesvirus phylogram (**a.1**) was based on the Jones Taylor Thornton gamma distributed with invariant sites model, while the gammaherpesvirus phylogram (**a.2**) was based on Le Gascuel gamma distributed with invariant sites model. (**B**) Maximum likelihood phylogram based on the Le Gascuel substation model with gamma distribution of the herpesvirus glycoprotein B deduced amino acid sequences (i) obtained in this study (green dots), (ii) the closest sequences to these sequences from GenBank, (iii) gammaherpesvirus sequences retrieved in other cetacean species, and (iv) gammaherpesvirus species recognized by the ICTV. *Human gammaherpesvirus 4*, *Human alphaherpesvirus 3* and *Human alphaherpesvirus 3* were selected as outgroup for alphaherpesvirus DPOL, gammherpesvirus DPOL and glycoprotein B phylograms. The reliability of the phylograms was tested via 1.000 bootstrap, and those bootstrap values lower than 70 were omitted.

In the DPOL tree of the alphaherpesvirus sequences, the sequences obtained in the Guiana dolphin MM672 and the Atlantic spotted dolphin ii230353 clustered with other cetacean alphaherpesviruses obtained in Delphinidae (Fig. 1a.1), while the alphaherpesvirus from the humpback whale clustered with an alphaherpesvirus obtained in a fin whale and with the *Beluga whale alphaherpesvirus 1*, the latter included within the genus *Varicellovirus* (Fig. 1a.1). None of the gammaherpesvirus DPOL sequences obtained in this study clustered with a bootstrap value over 70 with other gammaherpesvirus species (Fig. 1a.2). Regarding the gB phylogram, the gammaherpesvirus sequences obtained in two Atlantic spotted dolphins clustered with those from franciscanas and Bolivian river dolphins in the same subclade, with a bootstrap value of 100, and were classified into the genus *Rhadinovirus* (Fig. 1b). Of note, the other gB sequences from cetaceans were classified intro the genus *Bossavirus*.

### Gross and histopathological findings

None of the herpesvirus-positive animals presented tissue lesions consistent with those attributed to herpesvirus infection (Supplementary Table 2).

### Statistics

Region (south, southeastern, northeastern) and family (Delphinidae vs. Pontoporiidae) were the only independent variables for which statistically significant differences were observed. Specifically, the overall herpesvirus prevalence (those animals positive in liver and/or lung samples), the prevalence in liver and in lung were significantly higher in the southeastern region when compared to the northeastern region (p values of 0.0005, 0.0005 and 0.022, respectively). The family Pontoporiidae had higher herpesvirus prevalence than Delphinidae for the overall herpesvirus prevalence (p = 0.02) and the herpesvirus prevalence in liver samples (p = 0.0086). No statistically significant differences on the whole herpesvirus prevalence were found according to the type of tissue sample (p = 0.07).

## Discussion

To the authors’ knowledge, this is the largest study on herpesviruses in cetaceans of South America, a region with only a few herpesvirus descriptions in odontocetes—all of them in Brazil^32,34,44,45^. The herpesvirus overall prevalence (8.7%, 19/218) was similar to those described in cetaceans from the coast of Portugal (7.8%, 14/179)^46^, but apparently lower than the ones described in Ziphiidae of the Canary Islands in Spain (14.5%, 8/55)^47^ and in Delphinidae of the Spanish Cantabrian coast (78.6%, 11/14)^33^, which analyzed several tissue types by animal with the same DPOL PCR protocol used in the present study. However, ours was apparently higher than the prevalence described by Miyoshi et al.^48^ in several cetacean families from the coast of Japan (5.3%, 4/76). It is important to remark that, in our study, we combined two different PCR techniques for herpesvirus surveillance: the DPOL PCR described by VanDevanter et al.^49^ and the gB gene broad-spectrum PCR protocol of Ehlers et al.^50^—specific for gammaherpesvirus. The latter can be used successfully in cetaceans, being able to detect gammaherpesvirus in DPOL-negative cases, as observed in some franciscanas. The combination of DPOL and gB protocols increased our diagnostic sensitivity.

Regarding the anthropogenic activities, herpesvirus prevalence may be associated with the type of habitat, as coastal species are likely more exposed to immune impairments due to the multiple stressors present in this anthropized environment (e.g., pollution, infectious agents of terrestrial origin, habitat loss and degradation) and their narrow ecological niche^51^ than those from pelagic or mixed-habitat. This immunosuppression could play a role on the reactivation of herpesvirus^52^. However, we were not able to observe statistically significant differences regarding the prevalence of viral agents (in this case herpesvirus, the only one detected in the study) according to the type of habitat (coastal, mixed or pelagic). The most numerous coastal species analyzed were Guiana dolphins (n = 59) and franciscanas (n = 52), considered vulnerable to various impacts resulting from human activities^53,54^. Interestingly, the family Pontoporiidae (which comprises only one species, the franciscana) presented a significantly higher number of herpesvirus-positive individuals (p = 0.02) and of herpesvirus-positive liver samples when compared to Delphinidae included in the study. A previous article in this species that tested more tissues per individual also found a higher prevalence (14/27, 51.9%)^34^ - some of these animals were also included in our study. The reason for such high value in this threatened family—classified as Vulnerable by the IUCN Red List and as Critically Endangered by the Red Book of the Brazilian Fauna Threatened of Extinction^55,56^—is unknown, and the drivers should be identified. Of note, when compared to other groups, franciscanas form small groups -1 to 13 individuals^57^, which may limit herpesvirus transmission.

Although we did not detect differences in herpesvirus prevalence according to the habitat, a significantly higher HV prevalence was found in the southeastern when compared to the northeastern region. The southeastern region is the most human-impacted area of Brazil, and presents the largest port in Latin America^58^. Additionally, most of the sampled franciscanas—the species with the higher herpesvirus prevalence—stranded or were bycaught in the southeastern region, which may explain this difference.

There were no significant statistical differences regarding sex (p = 1.0) and age class (p = 0.68). Of note, seven out of 19 herpesvirus-positive individuals were calves (5 franciscanas and 2 Guiana dolphins, Table 2). In cetaceans, this age class counts with few herpesvirus descriptions, including an alphaherpesvirus infection in a beluga whale (*Delphinapterus leucas*) calf with malformation^59^ and another one in a sperm whale fetus (*Physeter macrocephalus*)^60^. We hypothesize that herpesvirus infection may occur in utero or during birth, as observed in other herpesviruses^61^. The lack of statistical differences among the studied age classes (adult, juvenile, calf) reinforces the vertical transmission hypothesis.

This study broadens the knowledge about the presence of herpesvirus in liver samples, a tissue with scarce descriptions of this agent^32,35,46,47,60,62–64^. The prevalence of herpesvirus in liver samples observed in our study (4.8%; 9/187) is similar to the one found in liver samples of Ziphiidae in the Canary Islands, Spain (2.8% (1/36)^47^, but apparently lower than the ones reported in this tissue in Delphinidae cetaceans from the Cantabrian and Mediterranean coasts of Spain (9.5%, 4/42 and 30%, 3/10, respectively)^35,62^. Of note, neither of the 30 liver samples of different cetacean families tested in Japan were positive^48^, nor the 14 liver samples of striped dolphins that stranded in the Cantabrian coast of Spain^33^.

Regarding lung samples, different herpesviruses have been detected in cetaceans in this tissue in previous studies^31,35,46–48,62,63,65^ and also in blowhole swabs^66^. The prevalence of herpesvirus in lung samples observed in our study (11.1%, 12/108) is similar to the one (10.4%, 5/48) reported in the same tissue in Ziphiidae by Felipe-Jiménez et al.^47^, apparently higher than those described in Delphinidae cetaceans from the Cantabrian (7.1%, 1/14) and Mediterranean coasts of Spain (7.5%, 4/53, and 15.4%, 2/13)^33,35,62^ and Italy (no detection in 11 lung samples tested)^67^, and in cetaceans of Japan (2.8%, 1/36)^48^.

None of the herpesvirus-positive individuals analyzed herein presented herpesvirus-associated lesions, as previously reported in other studies^33,34,46,48,62,67^. Nevertheless, it is not possible to rule out the pathogenicity of the detected viruses, especially due to the possibility of latent herpesvirus amplification. Of note, alphaherpesviruses and gammaherpesviruses have been associated with mucosal and cutaneous lesions in cetaceans^32,33,44,46,68^. Additionally, alphaherpesviruses have been associated with severe localized (e.g., nephritis, encephalitis and meningoencephalitis) and fatal systemic infections in cetaceans^31,47,59,63–65^. Further studies are required to elucidate if this viral family could (or not) cause hepatic and pulmonary lesions in cetaceans, as reported in other mammal taxa.

To the authors’ knowledge, this is the first report of herpesvirus in a pygmy sperm whale and a humpback whale worldwide. The obtained sequences were highly divergent when compared to those available in public genetic databases (GenBank/DDBJ/EMBL), and based on such findings and on their description in a novel host species, likely correspond to novel gammaherpesvirus species. We propose naming these strains Kogiid gammaherpesvirus 2 and Balaenopterid alphaherpesvirus 2; the herpesviral final species name should be formulated by the International Committee on Taxonomy of Viruses. These cetacean species belong to the families Kogiidae and Balaenopteridae, which count with few previous descriptions—two different alphaherpesvirus sequence types and a gammaherpesvirus in dwarf sperm whale^32,68^, and an alphaherpesvirus in a fin whale and two gammaherpesvirus sequence types in a common minke whale (*Balaenoptera acutorostrata*)^69^. The scarce number of previous descriptions is likely due to the limited number of virological studies performed in these pelagic animals, which should be prioritized.

The Gervais's beaked whale is an Atlantic species^70^. The high similarity observed between the alphaherpesvirus sequence type from the Gervais's beaked whale of Brazil and the one identified by Felipe-Jiménez et al.^47^ in another Gervais's beaked stranded in the Canary Islands may indicate that this species is the natural host of the detected herpesvirus, and that this virus may circulate along its distribution range; however, more reports are necessary to confirm or refute these hypotheses. Aside from these cases, there are only six other herpesvirus description in the genus *Mesoplodon*, i.e., a gammaherpesvirus found in a Stejneger's beaked whale (*Mesoplodon stejnegeri*) that stranded in Japan^48^, another gammaherpesvirus described in a Blainville’s beaked whale (*M. densirostris*) of the US with associated genital lesions^71,^ and in three Blainville’s beaked whales and a Sowerby’s beaked whale (*M. bidens*) of Spain—one of the former with associated interstitial nephritis^47,63^. Of note, in *Mesoplodon* the gammaherpesvirus found in a Blainville’s beaked whale (*M. densirostris*) was associated with genital lesions, while renal lesions (interstitial nephritis, tubuloepithelial necrosis, and the presence of intranuclear inclusions) have been associated with alphaherpesvirus in two Blainville’s beaked whales of Spain^47,63^. The remaining alpha- and gammaherpesviruses of Ziphiidae have not been directly associated with pathological changes. According to Rosel et al.^72^, there are 16 recognized species within the diverse genus *Mesoplodon*; therefore, it is expected to host a higher number of herpesvirus species.

The alphaherpesvirus obtained from Guiana dolphin MM672 was highly similar to others described in a short-beaked common dolphin from Portugal and two striped dolphins from the Mediterranean Sea (one from Italy and one from Spain), without associated histopathologic lesions^46,62,73^. All these species belong to the family Delphinidae. According to our findings and previous descriptions of similar sequences^46,73^, it is possible to suggest that they are different variants of the same alphaherpesvirus species, able to infect several cetacean species. The observed nt differences correspond to silent mutations when compared to those from Mediterranean striped dolphins, and could be due to the geographic distance between Brazil and Europe, or to the viral adaptation to different host species. This alphaherpesvirus is the second described in Guiana dolphins, a cetacean with previous reports of an alphaherpesvirus^32^ and a gammaherpesvirus^44^. The Guiana dolphin MM672 was coinfected with a cetacean poxvirus, amplified in a tattoo skin lesion^74^, being the first described case of a co-infection by herpesvirus and poxvirus in a cetacean in South America. Another Guiana dolphin (ii137962) included in our study likely presented a gammaherpesvirus (according to preliminary blast analysis), but the quality of the sequence was not adequate for further evaluation.

In Atlantic spotted dolphins, two different herpesviruses were found; (i) an alphaherpesvirus in Case ii230353, similar to those described in different Delphinidae species from the Mediterranean Sea and the Atlantic Ocean, and (ii) the same gammaherpesvirus gB sequence type in the kidney of case ii230353 and the lung of case ii213578, which presented a deduced aa sequence identical to Iniid gammaherpesvirus 1 (MZ209258), identified in Bolivian river dolphins^34^. In gammaherpesviruses retrieved from other mammals (felids and manatees), we have observed that the gB fragment obtained with the PCR protocols of Ehlers et al.^50^ is apparently more conserved than the DNA polymerase fragment amplified with the VanDevanter et al.^49^ protocol (unpublished data; ^75^), which may explain high similarity between gammaherpesviruses from Atlantic spotted dolphins and Bolivian river dolphins.

In franciscanas, we found a herpesvirus-positive case in Santa Catarina state, broadening the distribution range of the infectious agent in Brazil (until now, only described in São Paulo state^34^), and reinforcing the species potential role as a natural host for this infectious agent.

None of the tested liver and lung samples were positive to adenovirus or hepatitis E virus. In cetaceans, the knowledge about adenovirus is still very limited, restricted to previous molecular descriptions of novel adenovirus in feces of captive common bottlenose dolphins with self-limiting gastroenteritis or enteritis^76,77^, in lung samples of a wild adult male striped dolphin^41^, in the intestinal content, intestine and mesenteric lymph node of a wild harbor porpoise (*Phocoena phocoena*)^23^ and, recently, in spleen samples of six out of 59 (10.2%) wild bowhead whales (*Balaena mysticetus*) studied by De Luca et al.^24^. None of these cases had associated microscopic lesions. Regarding hepatitis E virus, none of the tested samples were positive. Until now, this agent had only been detected in common bottlenose dolphins with presumptive hepatitis kept in captivity in Cuba^18^, although it is commonly reported in the aquatic environment (e.g., in mussels)^78^. Recently, the exposure to hepatitis E virus was reported in captive and free-ranging cetaceans of Spain, although the infection was not molecularly confirmed^79^. The lack of adenovirus and hepatitis E virus detection in cetaceans of Brazil could be explained by (1) the absence of these agents, (2) low viral loads, or (3) lack of complementarity between the selected primers used in our broad spectrum PCR protocols and the cetacean adenoviruses and hepatitis E virus.

This study broadens the herpesvirus host range in cetaceans, including the first description in pygmy sperm whales and humpback whales. The prevalence of herpesvirus was significantly higher in the southeastern region of the country, the most industrialized of Brazil. Herpesvirus prevalence was significantly higher in Pontoporiidae family when compared to Delphinidae. Further studies are required to elucidate the drivers of herpesvirus prevalence in cetaceans.

## Materials and methods

### Case selection

For this study, we selected liver and lung samples of cetaceans (n = 218) that either stranded, were bycaught or found dead along the Brazilian coastline, between 2001 and 2021, inhabited coastal (n = 114), pelagic (n = 67) or mixed (which use both habitats, n = 37) habitats, and were in an adequate state of conservation at the time of sampling (codes 2 and 3, as described by Geraci and Lounsbury^80^). These animals were found in three different regions of Brazil (northeastern [states of Bahia and Ceará, n = 126], southeastern [states of Espírito Santo, Rio de Janeiro and São Paulo, n = 76] and southern [Santa Catarina state, n = 16]).

Overall, we analyzed 22 cetacean species that belonged to the families Delphinidae (n = 121), Pontoporiidae (n = 52), Balaenopteridae (n = 20), Kogiidae (n = 19), Physeteridae (n = 4), and Ziphiidae (n = 2). The families comprised species classified as coastal (Guiana dolphin [n = 59], franciscana [n = 52] and Lahille's bottlenose dolphin [*Tursiops truncatus gephyreus*, n = 3]—a coastal common bottlenose subspecies); mixed habitat (Bryde’s whale [*Balaenoptera brydei*, n = 2], humpback whale [n = 18], Atlantic spotted dolphin [n = 9], common dolphin [*Delphinus delphis*, n = 2], common bottlenose dolphin (*T. truncatus truncatus*, n = 2]), rough-toothed dolphin [*Steno bredanensis*, n = 2] and killer whale [*Orcinus orca*, n = 2]), and pelagic (Cuvier’s beaked whale [*Ziphius cavirostris*, n = 1], Gervais’ beaked whale [n = 1], pygmy sperm whale [n = 5], dwarf sperm whale [n = 14], short-finned pilot whale [*Globicephala macrorhynchus*, n = 8], melon-headed whale [*Peponocephala electra*, n = 9], pygmy killer whale [*Feresa attenuata*, n = 6], sperm whale [n = 4], Pantropical spotted dolphin [*Stenella attenuata*, n = 6], Clymene dolphin [*Stenella clymene*, n = 6], striped dolphin [n = 2], spinner dolphin [*Stenella longirostris*, n = 3], and Risso’s dolphin [*Grampus griseus*, n = 2]), based on Lodi and Borobia^81^.

Samples were obtained during necropsy procedures and preserved frozen at − 80 °C or RNAlater for molecular assays, and fixed in 10% buffered formalin solution and embedded in paraffin for histopathological examination. Age class (calf, juvenile, adult) was estimated according to total body length^81–84^, teeth histological analysis^85^ and/or features such as rostral hairs, and fetal line. Sex (male, female, unknown) was determined based on physical examination and/or gonadal evaluation. Overall, 93 cases were females, 124 males and one was undetermined. Regarding age class, we tested: 2 fetuses, 70 calves, 57 juveniles, and 89 adults.

In the studied cetaceans, DNA extractions were performed in species belonging to the families Delphinidae (n = 121), Pontoporiidae (n = 52), Balaenopteridae (n = 20), Kogiidae (n = 19), Physeteridae (n = 4), and Ziphiidae (n = 2), and RNA extractions were carried out in Delphinidae (n = 80), Pontoporiidae (n = 39), Balaenopteridae (n = 17), Kogiidae (n = 9), Physeteridae (n = 1), and Ziphiidae (n = 1) families. A detailed description of the tested cetacean families, species, and their classification as coastal, pelagic or mixed habitat is provided in Table 1.

**Table 1:** Family, species and origin of the cetacean species selected for this study, and liver and lung tested for the DNA viruses (herpesvirus and adenovirus) and RNA virus (hepatitis E virus) screening.

Family	Common name	Scientific name	Habitat	Number of tested animals			
				DNA viruses*			RNA virus**
				Liver	Lung	Overall	Liver
Balaenopteridae	Bryde's whale	*Balaenoptera brydei*	Mixed habitat	1	2	2	–
Balaenopteridae	Humpback whale	*Megaptera novaeangliae*	Mixed habitat	18	4	18	17
Physeteridae	Sperm whale	*Physeter macrocephalus*	Pelagic	3	3	4	1
Kogiidae	Pygmy sperm whale	*Kogia breviceps*	Pelagic	5	2	5	1
Kogiidae	Dwarf sperm whale	*Kogia sima*	Pelagic	12	10	14	8
Ziphiidae	Gervais's beaked whale	*Mesoplodon europaeus*	Pelagic	1	1	1	1
Ziphiidae	Cuvier's beaked whale	*Ziphius cavirostris*	Pelagic	1	1	1	–
Pontoporiidae	Franciscana	*Pontoporia blainvillei*	Coastal	33	26	52	39
Delphinidae	Short-beaked common dolphin	*Delphinus delphis*	Mixed habitat	2	0	2	–
Delphinidae	Pygmy killer whale	*Feresa attenuata*	Pelagic	6	4	6	3
Delphinidae	Short-finned pilot whale	*Globicephala macrorhynchus*	Pelagic	8	4	8	5
Delphinidae	Risso's dolphin	*Grampus griseus*	Pelagic	2	1	2	1
Delphinidae	Orca	*Orcinus orca*	Mixed habitat	2	1	2	2
Delphinidae	Melon-headed whalen	*Peponocephala electra*	Pelagic	6	7	9	8
Delphinidae	Guiana dolphin	*Sotalia guianensis*	Coastal	57	19	59	41
Delphinidae	Pantropical spotted dolphin	*Stenella attenuata*	Pelagic	6	5	6	6
Delphinidae	Clymene dolphin	*Stenella clymene*	Pelagic	6	5	6	5
Delphinidae	Striped dolphin	*Stenella coeruleoalba*	Pelagic	2	0	2	–
Delphinidae	Atlantic spotted dolphin	*Stenella frontalis*	Mixed habitat	7	7	9	5
Delphinidae	Spinner dolphin	*Stenella longirostris*	Pelagic	2	2	3	2
Delphinidae	Rough-toothed dolphin	*Steno bredanensis*	Mixed habitat	2	0	2	–
Delphinidae	Common bottlenose dolphin	*Tursiops truncatus truncatus*	Mixed habitat	2	2	2	2
Delphinidae	Lahille’s bottlenose dolphin	*Tursiops truncatus gephyreus*	Coastal	3	2	3	–
	Total			187	108	218	147

*Adenovirus and herpesvirus.

**Hepatitis E virus.

### Molecular methods

We selected samples from 218 different cetaceans (187 liver and 108 lung samples) to survey selected DNA virus (adenovirus and herpesvirus), and from 147 cetaceans (147 liver samples) for RNA virus (hepatitis E virus) (Table 1).

A targeted and systematic search for selected infectious agents (herpesvirus, adenovirus and HEV) was performed using molecular diagnostics. Frozen liver samples were manually or automatically homogenized, digested for 24 h with proteinase K (for DNA extraction), and centrifuged at 5.000 rpm for 5 min. Subsequently, the supernatant was extracted using commercial DNA or RNA kits (DNeasy Blood & Tissue kit and RNeasy mini kit, Qiagen, Hilden, Germany), according to the manufacturers' instructions. Samples preserved in RNAlater were washed with PBS before the extraction of the nucleic acids. Aside from liver and/or lung samples, which were selected for the initial screening, other tissue samples available from cases positive to the selected agents that were kept frozen or in RNAlater were also extracted and tested (Table 2). All DNA-extracted samples were tested for herpesvirus (DNA polymerase [DPOL] and glycoprotein B [gB] genes) and adenovirus (DPOL gene) by nested broad spectrum PCR techniques, while RNA extractions were tested for hepatitis E virus (open reading frame 1 of the RNA-dependent RNA polymerase region) using a nested PCR technique with an initial reverse transcription step^34,49,50,86–88^; Supplementary Table 1). Of note, the DPOL PCR protocol is able to amplify alpha-, beta- and gammaherpesvirus, while the gB PCR is specific to gammaherpesvirus^49,50^. As positive controls, we selected positive samples to Magellanic penguin alphaherpesvirus (HV DPOL protocol), giant armadillo gammaherpesvirus (HV DPOL and gB protocols), fur seal gammaherpesvirus (HV gB protocol), a vaccine of canine adenovirus type 2 (adenovirus DPOL protocol), and a pig sample positive for hepatitis E genotype 3 (hepatitis E virus “open reading frame 1” protocol). DPEC water was selected as non-template control.

**Table 2:** Identification number, species, sex, age class, date and stranding location of the cetaceans with liver and lung samples positive to the DNA polymerase (DPOL) and glycoprotein B (gB) PCR protocols.

ID Nº	Species	Sex	Age class	Stranding date (DD/MM/YYYY)	Local	Tissues tested				
						Liver		Lung		Other tissues (positive tissues marked in bold)
						DPOL	gB	DPOL	gB	
MM579*	*Kogia sima*	M	J	04/05/2014	Santos—SP	+ (α)	–	+ (α)	–	Cerebellum, cerebrum, epididymis, esophagus, eye, heart, intestine (DPOL), kidney, mesenteric lymph node (DPOL), stomach (DPOL), skin (DPOL), spinal cord, testicle, tongue, trachea
MM672	*Sotalia guianensis*	M	J	17/10/2009	Rio de Janeiro—RJ	+ (α)	–	NT	NT	–
MM731*	*Sotalia guianensis*	F	C	06/01/2015	Linhares—ES	+ (α)	–	NT	NT	Blood (DPOL), kidney (DPOL), muscle, skin (DPOL)
ii94246	*Pontoporia blainvillei*	M	A	12/09/2018	Praia Grande—SP	–	+ (γ)	NT	NT	–
ii126142	*Pontoporia blainvillei*	F	C	02/11/2018	Ilha Comprida—SP	–	+ (γ)	+ (γ)	+ (γ)	Brain stem, duodenum (DPOL, gB), kidney (gB), thymus (DPOL, gB)
ii151268**	*Pontoporia blainvillei*	F	C	29/11/2019	Itanhaém—SP	+ (γ)	–	NT	NT	–
ii160187	*Pontoporia blainvillei*	M	A	07/12/2019	Praia Grande—SP	–	+ (γ)	–	–	Brain stem, kidney, mesenteric lymph node (DPOL), spleen (DPOL)
ii166901**	*Pontoporia blainvillei*	F	A	17/03/2020	Praia Grande—SP	+ (γ)	+ (γ)	NT	NT	–
ii179266	*Kogia breviceps*	F	A	22/10/2019	Laguna—SC	+ (γ)	–	NT	NT	Cerebellum, kidney, mesenteric lymph node, spleen (DPOL)
MM332**	*Pontoporia blainvillei*	M	C	23/06/2011	Praia Grande—SP	NT	NT	–	+ (γ)	Blood (gB), mesenteric lymph node, spleen
MM395**	*Pontoporia blainvillei*	M	J	22/10/2001	Ubatuba—SP	–	–	–	+ (γ)	–
MM525	*Pontoporia blainvillei*	M	C	11/04/2013	São Francisco do Sul—SC	NT	NT	–	+ (γ)	Brain, blood, mesenteric lymph node (DPOL gB)
MM550**	*Pontoporia blainvillei*	M	J	06/11/2013	Praia Grande, SP	NT	NT	–	+ (γ)	Mesenteric lymph node (DPOL), prescapular lymph node, testicle (DPOL, gB)
MM568**	*Pontoporia blainvillei*	M	C	31/01/2014	Guarujá—SP	NT	NT	+ (γ)	+ (γ)	Adrenal gland, heart, kidney, mesenteric lymph node, prescapular lymph node (DPOL, gB), skeletal muscle (DPOL, gB), skin (gB), thymus (gB)
MM382	*Mesoplodon europaeus*	F	A	27/01/2011	Cruz—CE	–	–	+ (α)	–	Heart (DPOL), intestine, kidney, placenta, spleen (DPOL), stomach, uterus
ii137962	*Sotalia guianensis*	F	C	03/03/2019	Cananéia—SP	–	–	+ (γ)	–	Brain stem, kidney, mesenteric lymph node, spleen, thymus
ii213578	*Stenella frontalis*	M	A	20/08/2020	São Francisco do Sul—SC	–	–	–	+ (γ)	Brain stem, kidney, mesenteric lymph node, spleen
ii230353	*Stenella frontalis*	F	J	21/11/2020	Balneário Barra do Sul—SC	–	–	+ (α)	–	Brain stem, kidney (gB)***, mesenteric lymph node, spleen
Mn939	*Megaptera novaeangliae*	M	J	3/08/2018	Aracruz—ES	–	–	+ (α)	–	Duodenum

*CE* Ceará state, *ES* Espírito Santo state, *RJ* Rio de Janeiro state, *SC* Santa Catarina state, *SP* São Paulo state, *M* male, *F* female, *C* calf, *J* juvenile, *A* adult. The gammaherpesvirus subfamilies found in the positive samples are indicated with α (*Alphaherpesvirinae*) and γ (*Gammaherpesvirinae*).

*Cases previously published by Sacristán et al.^32^, presenting two different alphaherpesvirus species, one in intestine and skin, and another in stomach and mesenteric lymph node.

**Cases previously published by Exposto Novoselecki et al.^34^.

***The kidney of ii230353 presented a gammaherpesvirus.

Those samples positive to any of the selected viruses were purified using the ExoSap-IT reagent (USB Corporation, Ohio, USA) and confirmed by direct Sanger sequencing of the amplicons in both directions in the ABI 3730 DNA Analyzer. The obtained sequences were aligned using the Muscle algorithm in MEGA Software 7.0 program^89^ to build consensus sequences. Once the primers were eliminated, the consensus sequences were compared with those available in GenBank using the BLAST algorithm, and the percentage of identity among them was determined based on the distance p ([1 – p-distance)*100]). Finally, we used the MEGA 7.0 program to perform the alignments with other sequences of the same viral family and build the phylograms, selecting 1000 bootstraps. The evolutionary models selected for the amino acid (aa) phylograms were: Le and Gascual with invariants sites (LG + I) for the alphaherpesvirus DNA polymerase tree, Jones-Taylor-Thornton with invariants sites and gamma distribution (JTT + I + G) for the gammaherpesvirus DNA polymerase tree, and LG + I + G for the glycoprotein B tree, according to the results of ProtTest analyses.

In this study, we included some liver and/or lung herpesvirus-positive animals that had been previously published by our group, in order to evaluate and compare the prevalence of the herpesvirus in the different tested species of Brazil (Table 2). Specifically, the DPOL sequences found in the liver and lung of the dwarf sperm whale MM579, and in the liver of the herpesvirus-positive Guiana dolphin MM731, correspond to alphaherpesviruses, and were previously described by Sacristán et al.^32^. Additionally, six franciscana cases (MM332, MM395, MM550, MM568, ii151268 and ii166901) were previously published by Exposto Novoselecki et al.^34^ as gammaherpesvirus-positive, with 2 DPOL and 1 gB sequence found in their livers, and 1 DPOL and 4 gB sequences detected in their lungs (Table 2). The remaining herpesvirus-positive cases were sequenced.

### Histopathology

All available liver and lung samples of the animals positive to the selected viruses were microscopically evaluated to establish a potential “cause-effect” diagnostic between the presence of an identified infectious agent and the morphology of the observed lesion. In addition, the remaining available tissues of the positive cases were also histologically evaluated. All tissue samples fixed in 10% formalin were processed with routine techniques of paraffin embedding, cut at 5 µm and stained using the hematoxylin–eosin technique.

### Statistics

Following the analysis of the selected agents, we performed conventional statistic tests (Chi square, Kruskal–Wallis) in the R program^90^ to elucidate if the prevalence (dependent variable) of the detected viruses could be associated with the following variables related to the individual’s history (independent variables): species habitat (costal, pelagic or mixed habitat), region (northeastern, southeastern, south), sex (male, female), age class (calf, juvenile, adult), and family (in those with more than ten individuals). The statistical evaluation was performed based on herpesvirus results obtained in (i) the total number of animals tested (in liver and/or lung), (ii) the total number of liver samples tested and (iii) the total number of lung samples analyzed. Only those variables with a p value < 0.05 were considered significant.

### Permits

All studied samples were collected in full compliance with specific federal permits issued by the System of Authorization and Information on Biodiversity of the Chico Mendes Institute for Biodiversity Conservation (ICMBio) (SISBIO, license No 72608-1) and by the Brazilian Ministry of Environment and Climate Change (ABIO no. 1169/2019; SISGEN authorization no. AAF009C). All procedures were performed in accordance with the Ethics Committee in the Use of Animals (CEUA) of the School of Veterinary Medicine and Animal Sciences, University of São Paulo (Process number No 7151291019). No experiments were performed on live animals.

### Ethical standards

All studied samples were collected in full compliance with specific federal permits issued by the System of Authorization and Information on Biodiversity (SISBIO) of the Chico Mendes Institute for Biodiversity Conservation (ICMBio, license No 72608-1), and by the Brazilian Ministry of Environment (ABIO no. 1169/2019). All procedures were performed in accordance with the Ethics Committee in the Use of Animals—CEUA) (certificate No 7151291019) of the School of Veterinary Medicine and Animal Science, University of São Paulo (FMVZ-USP). Part of the Samples from São Paulo and Santa Catarina states were collected as part of the Santos Basin Beach Monitoring Project (Projeto de Monitoramento de Praias da Bacia de Santos—PMP-BS), licensed by the Brazilian Institute of the Environment and Renewable Natural Resources (IBAMA) of the Brazilian Ministry of Environment (ABIO No 1169/2019). ARRIVE guidelines: not applicable.

### Supplementary Information

Supplementary Tables.

## Data Availability

All data are available in the manuscript. The datasets generated and/or analyzed during the current study are available in the GenBank repository (accession numbers OQ980334 to OQ980338, OQ926585 and OQ926586).

## References

[1] Trites AW, Deecke VB, Gregr EJ, Ford JK, Olesiuk PF (2007). Killer whales, whaling, and sequential megafaunal collapse in the north Pacific: A comparative analysis of the dynamics of marine mammals in Alaska and British Columbia following commercial whaling. Mar. Mamm. Sci..

[2] Bossart G (2011). Marine mammals as sentinel species for oceans and human health. Vet. Pathol..

[3] Bejder L (2006). Decline in relative abundance of bottlenose dolphins exposed to long-term disturbance. Conserv. Biol..

[4] Azevedo AF (2017). The first confirmed decline of a delphinid population from Brazilian waters: 2000–2015 abundance of *Sotalia guianensis* in Guanabara Bay, South-eastern Brazil. Ecol. Indic..

[5] Van Bressem MF (2009). Emerging infectious diseases in cetaceans worldwide and the possible role of environmental stressors. Dis. Aquat. Organ..

[6] Jackson JB (2010). The future of the oceans past. Philos. Trans. R. Soc. Lond. B Biol. Sci..

[7] Gulland FMD, Hall AJ, Reynolds JE, Perrin WF, Reeves RR, Montgomery S, Ragen T (2006). The role of infectious disease in influencing status and trends. Marine Mammal Research: Conservation Beyond Crisis.

[8] Sweeney JC, Fowler ME (1978). Noninfectious diseases. Zoo and Wild Animal Medicine.

[9] Baker JR (1992). Causes of mortality and parasites and incidental lesions in dolphins and whales from British waters. Vet. Rec..

[10] Venn-Watson S, Daniels R, Smith C (2012). Thirty year retrospective evaluation of pneumonia in a bottlenose dolphin *Tursiops truncatus* population. Dis. Aquat. Organ..

[11] Pierce GJ (2008). Bioaccumulation of persistent organic pollutants in female common dolphins (*Delphinus delphis*) and harbour porpoises (*Phocoena phocoena*) from western European seas: geographical trends, causal factors and effects on reproduction and mortality. Environ. Pollut..

[12] Smith CR (2012). Pulmonary ultrasound findings in a bottlenose dolphin *Tursiops truncatus* population. Dis. Aquat. Organ..

[13] Terracciano G (2020). Dolphins stranded along the tuscan coastline (Central Italy) of the "Pelagos Sanctuary": A parasitological investigation. Pathogens.

[14] Dailey MD, Dierauf LA, Gulland FMD (2001). Parasitic diseases. CRC Handbook of Marine Mammal Medicine.

[15] Resendes AR (2002). Hepatic sarcocystosis in a striped dolphin (*Stenella coeruleoalba*) from the Spanish Mediterranean coast. J. Parasitol..

[16] Gonzales-Viera O (2013). Toxoplasmosis in a Guiana dolphin (*Sotalia guianensis*) from Paraná, Brazil. Vet. Parasitol..

[17] Dotzauer A, Feinstone SM, Kaplan G (1994). Susceptibility of nonprimate cell lines to hepatitis A virus infection. J. Virol..

[18] Montalvo Villalba MC (2017). Hepatitis E virus in bottlenose dolphins *Tursiops truncatus*. Dis. Aquat. Organ..

[19] Mihindukulasuriya KA, Wu G, St Leger J, Nordhausen RW, Wang D (2008). Identification of a novel coronavirus from a beluga whale by using a panviral microarray. J. Virol..

[20] Sierra E (2014). Fatal systemic morbillivirus infection in bottlenose dolphin, Canary Islands, Spain. Emerg. Infect. Dis..

[21] Pei C (2012). Herpes-like virus infection in Yangtze finless porpoise (*Neophocaena phocaenoides*): Pathology, ultrastructure and molecular analysis. J. Wild. Dis..

[22] Kennedy-Stoskopf S, Dierauf LA, Gulland FMD (2001). Viral diseases. CRC Handbook of Marine Mammal Medicine.

[23] van Beurden SJ (2017). A novel cetacean adenovirus in stranded harbour porpoises from the North Sea: Detection and molecular characterization. Arch. Virol..

[24] De Luca E, Stimmelmayr R, Rotstein DS, Sanchez S (2021). A Novel adenovirus detected in Bering-Chukchi-Beaufort seas bowhead whale (*Balaena mysticetus*): Epidemiologic data and phylogenetic characterization. J. Wildl. Dis..

[25] Tryland M, Larsen AK, Nymo I, Gulland FMD, Dierauf AL, Whitman KL (2018). Bacterial infections and diseases. CRC Handbook of Marine Mammal Medicine.

[26] Guimarães JP, Febronio AM, Vergara-Parente JE, Werneck MR (2015). Lesions associated with *Halocercus*
*brasiliensis* Lins de Almeida, 1933 in the lungs in the lungs of dolphins stranded in the Northeast of Brazil. J. Parasitol..

[27] Groch KR (2018). Pulmonary and systemic fungal infections in an Atlantic spotted dolphin and a Bryde's whale, Brazil. Dis. Aquat. Organ..

[28] Costa-Silva S (2019). *Toxoplasma gondii* in cetaceans of Brazil: A histopathological and immunohistochemical survey. Braz. J. Vet. Parasitol..

[29] Lipscomb TP (1996). Morbilliviral epizootic in bottlenose dolphins of the Gulf of Mexico. J. Vet. Diagn. Invest..

[30] Anthony SJ (2013). Identification of a novel cetacean polyomavirus from a common dolphin (*Delphinus delphis*) with tracheobronchitis. PloS One.

[31] Blanchard TW (2001). Two novel alphaherpesviruses associated with fatal disseminated infections in Atlantic bottlenose dolphins. J. Wildl. Dis..

[32] Sacristán C (2019). Novel herpesviruses in riverine and marine cetaceans from South America. Acta Trop..

[33] Vargas-Castro I (2020). Alpha- and gammaherpesviruses in stranded striped dolphins (*Stenella coeruleoalba*) from Spain: First molecular detection of gammaherpesvirus infection in central nervous system of odontocetes. BMC Vet. Res..

[34] Exposto Novoselecki H (2021). Highly divergent herpesviruses in threatened river dolphins from Brazil. Sci. Rep..

[35] Vargas-Castro I (2021). Systematic determination of herpesvirus in free-ranging cetaceans stranded in the western mediterranean: Tissue tropism and associated lesions. Viruses.

[36] Nollens HH (2008). Characterization of a parainfluenza virus isolated from a bottlenose dolphin (*Tursiops truncatus*). Vet. Microbiol..

[37] Osterhaus AD (1985). The isolation and partial characterization of a highly pathogenic herpesvirus from the harbor seal (*Phoca vitulina*). Arch. Virol..

[38] Kleiboeker SB (2002). Association of two newly recognized herpesviruses with interstitial pneumonia in donkeys (*Equus asinus*). J. Vet. Diagn. Invest..

[39] Jaber JR (2004). Hepatic lesions in cetaceans stranded in the Canary Islands. Vet. Pathol..

[40] Di Guardo G (1995). Post mortem investigations on cetaceans found stranded on the coasts of Italy between 1990 and 1993. Vet. Rec..

[41] Giorda F (2017). Postmortem findings in cetaceans found stranded in the Pelagos Sanctuary, Italy, 2007–21. J. Wild. Dis..

[42] Harvell CD (1999). Emerging marine diseases–climate links and anthropogenic factors. Science.

[43] Acevedo-Whitehouse K, Duffus AL (2009). Effects of environmental change on wildlife health. Philos. Trans. R. Soc. Lond. B Biol. Sci..

[44] Seade GCC (2017). Herpesviral infection in a Guiana dolphin (*Sotalia guianensis*) from the northern coast of Brazil. J. Vet. Diagn. Invest..

[45] Costa-Silva S (2023). Short-finned pilot whale strandings associated with pilot whale morbillivirus, Brazil. Emerg. Infect. Dis..

[46] Bento MC (2019). Herpesvirus infection in marine mammals: A retrospective molecular survey of stranded cetaceans in the Portuguese coastline. Infect. Genet. Evol..

[47] Felipe-Jiménez I (2021). Contribution to herpesvirus surveillance in beaked whales stranded in the Canary Islands. Animals.

[48] Miyoshi K (2011). Molecular identification of novel alpha- and gammaherpesviruses from cetaceans stranded on Japanese coasts. Zoolog. Sci..

[49] VanDevanter DR (1996). Detection and analysis of diverse herpesviral species by consensus primer PCR. J. Clin. Microbiol..

[50] Ehlers B (2008). Novel mammalian herpesviruses and lineages within the Gammaherpesvirinae: Cospeciation and interspecies transfer. J. Virol..

[51] Reeves RR, Smith BD, Crespo EA, di Sciara GN (2003). Dolphins, Whales and Porpoises: 2002–2010 Conservation Action Plan for the World’s Cetaceans.

[52] Woźniakowski G, Samorek-Salamonowicz E (2015). Animal herpesviruses and their zoonotic potential for cross-species infection. Ann. Agric. Environ. Med..

[53] Domit C, Simões-Lopes PC, Cremer MJ (2022). Chapter 12—Coastal development and habitat loss: understanding and resolving associated threats to the franciscana, *Pontoporia blainvillei*. The Franciscana Dolphin on the Edge of Survival.

[54] Soares ED, Cantor M, Bracarense APFRL, Groch KR, Domit C (2022). Health conditions of Guiana dolphins facing cumulative anthropogenic impacts. Mammal. Biol..

[55] MMA (Ministério de Medio Ambiente). Lista Nacional Oficial de Espécies da Fauna Ameaçadas de Extinção – Mamíferos, Aves, Répteis, Anfíbios e Invertebrados Terrestres. Portaria MMA no. 444, de 17 de dezembro de 2014 Brazil. http://www.icmbio.gov.br/cepsul/images/stories/legislacao/Portaria/2014/p_mma_444_2014_lista_esp%C3%A9cies_ame%C3%A7adas_extin%C3%A7%C3%A3o.pdf (2014).

[56] Zerbini, A. N., Secchi, E., Crespo, E., Danilewicz, D. & Reeves, R. *Pontoporia blainvillei* (errata version published in 2018). *The IUCN Red List of Threatened Species*10.2305/IUCN.UK.2017-3.RLTS.T17978A50371075.en.

[57] Secchi ER, Cremer MJ, Danilewicz D, Lailson-Brito J (2021). A synthesis of the ecology, human-related threats and conservation perspectives for the endangered Franciscana dolphin. Front. Mar. Sci..

[58] Ewbank AC (2022). World Health Organization critical priority *Escherichia coli* clone ST648 in magnificent frigatebird (*Fregata magnificens*) of an uninhabited insular environment. Front. Microbiol..

[59] Burek-Huntington KA (2022). Congenital defects and herpesvirus infection in beluga whale *Delphinapterus leucas* calves from the critically endangered cook inlet population. Dis. Aquat. Organ..

[60] Mazzariol S (2018). Multidisciplinary studies on a sick-leader syndrome-associated mass stranding of sperm whales (*Physeter macrocephalus*) along the Adriatic coast of Italy. Sci. Rep..

[61] James SH, Sheffield JS, Kimberlin DW (2014). Mother-to-child transmission of herpes simplex virus. J. Pediatric Infect. Dis. Soc..

[62] Bellière EN (2010). Presence of herpesvirus in striped dolphins stranded during the cetacean morbillivirus epizootic along the Mediterranean Spanish coast in 2007. Arch. Virol..

[63] Arbelo M (2012). Herpes virus infection associated with interstitial nephritis in a beaked whale (*Mesoplodon densirostris*). BMC Vet. Res..

[64] Sierra E (2022). Molecular characterization of herpesviral encephalitis in cetaceans: Correlation with histopathological and immunohistochemical findings. Animals.

[65] Arbelo M (2010). Herpesvirus infection with severe lymphoid necrosis affecting a beaked whale stranded in the Canary Islands. Dis. Aquat. Organ..

[66] IJsseldijk, L. L.  (2018). Beached bachelors: An extensive study on the largest recorded sperm whale *Physeter macrocephalus* mortality event in the North Sea. PLoS One.

[67] Lecis R (2014). First Gammaherpesvirus detection in a free-living Mediterranean bottlenose dolphin. J. Zoo Wildl. Med..

[68] Smolarek Benson KA (2006). Identification of novel alpha- and gammaherpesviruses from cutaneous and mucosal lesions of dolphins and whales. J Virol. Methods.

[69] Melero M, Crespo-Picazo JL, Rubio-Guerri C, García-Párraga D, Sánchez-Vizcaíno JM (2015). First molecular determination of herpesvirus from two mysticete species stranded in the Mediterranean Sea. BMC Vet. Res..

[70] Reeves RR, Folkens PA (2002). Guide to Marine Mammals of the World.

[71] Saliki JT (2006). A novel gammaherpesvirus associated with genital lesions in a Blainville’s beaked whale (*Mesoplodon densirostris*). J. Wildl. Dis..

[72] Rosel, P. E., Archer, F. I., Baker, C. S., & Boness, D. J. List of marine mammal species and subspecies. Committee on Taxonomy, The Society for Marine Mammalogy. https://marinemammalscience.org/science-and-publications/list-marine-mammal-species-subspecies/ (2019).

[73] Grattarola C (2018). Occlusive mycotic tracheobronchitis and systemic Alphaherpesvirus coinfection in a free-living striped dolphin *Stenella coeruleoalba* in Italy. Dis. Aquat. Organ..

[74] Sacristán C (2018). Molecular identification and microscopic characterization of poxvirus in a Guiana dolphin and a common bottlenose dolphin, Brazil. Dis. Aquat. Organ..

[75] Ewbank AC (2023). Herpesvirus and adenovirus surveillance in threatened wild West Indian (*Trichechus manatus*) and Amazonian manatees (*Trichechus inunguis*). Brazil. Acta Trop..

[76] Rubio-Guerri C (2015). Novel adenovirus detected in captive bottlenose dolphins (*Tursiops truncatus*) suffering from self-limiting gastroenteritis. BMC Vet. Res..

[77] Standorf K (2018). Phylogenetic analysis of the genome of an enteritis-associated bottlenose dolphin Mastadenovirus supports a clade infecting the Cetartiodactyla. J. Wild. Dis..

[78] Crossan C (2012). Hepatitis E virus genotype 3 in shellfish, United Kingdom. Emerg. Infect. Dis..

[79] Caballero-Gómez J (2022). Hepatitis E virus infections in free-ranging and captive cetaceans, Spain, 2011–2022. Emerg. Infect. Dis..

[80] Geraci JR, Lounsbury VJ (2005). Marine Mammals Ashore: A Field Guide for Strandings.

[81] Lodi L, Borobia M (2013). Baleias botos e golfinhos do Brasil, guia de identificação.

[82] Di Beneditto APM, Arruda Ramos RM (2001). Biology and conservation of the franciscana (Pontoporia blainvillei) in the north of Rio de Janeiro State, Brazil. J. Cetacean Res. Manag..

[83] Rosas FCW, Monteiro-Filho ELA (2002). Reproduction of the Estuarine Dolphin (*Sotalia guianensis*) on the Coast of Paraná, Southern Brazil. J. Mammal.

[84] Rosas FCW, Monteiro-Filho ELA (2002). Reproductive parameters of *Pontoporia blainvillei* (Cetacea, Pontoporiidae), on the coast of São Paulo and Paraná States, Brazil. Mammalia.

[85] Hohn AA, Leatherwood S, Reeves RR (1990). Reading between the lines: Analysis of age estimation in dolphins. The Bottlenose Dolphin.

[86] Johne R (2010). Detection of a novel hepatitis E-like virus in faeces of wild rats using a nested broad-spectrum RT-PCR. J. Gen. Virol..

[87] Li Y (2010). Host range, prevalence, and genetic diversity of adenoviruses in bats. J. Virol..

[88] Lial HC (2022). Adenovirus surveillance in wild carnivores from Brazil. Infect. Genet. Evol..

[89] Kumar S, Stecher G, Tamura K (2016). MEGA7: Molecular evolutionary genetics analysis version 7.0 for bigger datasets. Mol. Biol. Evol..

[90] R Core Team. 2014. R: A language and environment for statistical computing [Computer software]. Vienna, Austria: R Foundation for Statistical Computing. http://www.R-project.org/.

