# Supplementary material for: Survey of selected viral agents (herpesvirus, adenovirus and hepatitis E virus) in liver and lung samples of cetaceans, Brazil
Source: Sci Rep. 2024 Feb 1;14:2689. doi: 10.1038/s41598-023-45315-9 (PMC10834590; doi:10.1038/s41598-023-45315-9)
Supplement: Supplementary file 1 — Supplementary Tables. [file 41598_2023_45315_MOESM1_ESM.docx]

**SUPPLEMENTAY FILES**

**Supplementary table 1.** PCR techniques selected to detect herpesvirus, adenovirus and hepatitis E virus (HEV).

| **Genetic region of interest** | **Agent** | **Technique** | **Reference** |
| --- | --- | --- | --- |
| DNA polymerase | Herpesvirus | Broad spectrum nested PCR | VanDevanter et al. 1996 |
| Glycoprotein B |  | Broad spectrum nested PCR | Ehlers et al. 2008, Exposto Novoselecki et al. 2021 |
| DNA polymerase | Adenovirus | Broad spectrum nested PCR | Li et al. 2010, Lial et al. 2022 |
| ORF1* | HEV | Broad spectrum nested PCR with reverse transcription | Johne et al. 2010 |

*ORF1 = open reading frame 1 of the RNA-dependent RNA polymerase region.

**Supplementary Table 2.** Gross and histopathologic findings of the herpesvirus-positive cetaceans.

| **Case nº** | **Gross findings** | **Histopathologic findings** |
| --- | --- | --- |
| MN939 | Absent macroscopic description. | **Small intestine, associated lymphoid tissue, adjacent smooth muscle, pancreas:** advanced autolysis; presence of large quantity of bacterial colonies. |
| MM672 | **Skin**: tattoo skin lesions: enlarged and coalescent (4x3cm). Other similar round lesions observed on R lateral flank (2x2cm) and blowhole (4x3cm). Net entanglement marks. | **Skeletal muscle, heart:** advanced autolysis, without alterations consistent with inflammation/infectious process; **Liver:** advanced autolysis. |
| ii230353 | **Lung**: hyperinflated left lung, moderate-severe pulmonary congestion.  **Skin**: multifocal marks consistent with fishing interaction.  **Uterus**: uterine congestion. | **Lung:** moderate congestion, mild multifocal hemorrhage, mainly peribronchial, emphysema, presence of putrefaction bacteria. **Kidney:** mild congestion multifocal, presence of few secondary cysts. **Liver:** mild multifocal mixed periportal hepatitis. **Lymph node:** marked germinal centers’ distension and follicular center lymphocytolysis, presence of mild to moderate quantity of granulocytes in perifollicular region. **Thymus:** presence of Hassall’s corpuscles, preserved lymphoid tissue, but with autolysis.  **Adrenal gland:** moderate autolysis, NSFO. **Heart:** NSFO. **Skeletal muscle:** NSFO. **Skin:** NSFO. **Not specified** **mucosa:** NSFO. |
| ii160187 | **Esophagus**: mild generalized esophagitis.  **Lung**: severe generalized pulmonary congestion and mild presence of multifocal nodules.  **Skin**: entanglement marks on dorsal and caudal fin. Interspecific marks throughout the body, scars in caudal, dorsal and pectoral fins.  **Stomach**: moderate-mild generalized gastritis. | **Adrenal** **gland:** mild congestion. **Cerebrum:** mild congestion. **Colon:** mild, diffuse eosinophilic colitis. **Kidney:** moderate congestion associated to mild hemorrhagic foci, mild multifocal proliferative glomerulonephritis, mild diffuse tubular degeneration. **Large** **intestine:** mild diffuse mixed enteritis, with multifocal formation of cryptal microabscesses, focal dystrophic calcification, lymphoid expansion associated to lymphocytolysis. **Liver** – mild multifocal to coalescent congestion, mild diffuse macro and microgoticular degeneration. **Lung:** marked diffuse congestion, associated to mild to moderate multifocal areas of intra-alveolar hemorrhage, multifocal presence of intra-alveolar oval basophilic structures, with approximately 1 mm of the major axis, mild to moderate quantity of alveolar macrophages, presence of few intra-alveolar granulocytes. **Lymph node 1:** marked germinate proliferation, with paracortical expansion, mild granulocytic splenitis, congestion moderate. **Lymph node 2:** mild multifocal a coalescent granulocytic lymphadenitis, lymphoid expansion and pericapsular edema. **Lymph node 3:** mild, multifocal a coalescent granulocytic lymphadenitis, lymphoid expansion. **Pancreas:** partially autolyzed, mild congestion. **Perivesical ligaments:** mild focal hemorrhage, moderate congestion. **Skin:** mild congestion. **Trachea:** partially autolyzed, mild to moderate congestion.  **Cerebellum:** NSFO. **Heart:** NSFO. **Skeletal muscle:** NSFO. **Stomach:** partially autolyzed. **Urinary bladder:** NSFO. |
| ii126142 | Fetal folds, umbilical cord, folded dorsal and caudal fins and vibrissae, indicating recent birth. Cervical/ventral hematoma. Mild focal submandibular hematoma.  **Eye**: erythematous OD conjunctiva.  **Brain**: cerebral edema.  **Heart**: moderate generalized myocardial hypertrophy.  **Kidney**: moderate diffuse renal congestion with altered cortico-medullar relationship.  **Liver**: neonatal hepatic steatosis.  **Lung**: moderate generalized pulmonary edema, congestion and atelectatic pulmonary parenchyma.  **Thymus:** moderate generalized congestion, lymphadenopathy, mild diffuse splenic congestion. | **Adrenal gland:** mild congestion and focal areas of cortical mild hemorrhage. **Heart:** mild diffuse congestion, mild diffuse granulocytic myocarditis, mild diffuse degeneration and disruption of the cardiac fibers. **Kidney:** mild multifocal congestion, micro/macrogoticular tubular degeneration. **Liver:** mild diffuse congestion, moderate diffuse microgoticular degeneration, mild multifocal hyperplasia ductal, mild multifocal to coalescent loss of hepatocyte cord architecture associated to scarce granulocytes. **Lung:** marked congestion, associated to desquamation of epithelial cells and macrophages, mild presence of intra-alveolar hyaline membranes, presence of rare intra-alveolar ciliated cellular structures (protozoans), presence of rare granulocytes.  **Thymus:** mild to moderate congestion, marked lymphoid proliferation, associated to areas of lymphocytolysis, large quantity of Hassall’s corpuscles, with presence of associated giant cells.  **Colon:** NSFO. **Cerebral cortex:** NSFO. **Fragment of CNS:** partially autolyzed, NSFO. **Skeletal muscle:** NSFO. |
| ii213578 | **Cerebral meninges**: meningeal congestion.  **Heart**: moderate to marked endocardiosis in mitral and tricuspid cardiac valves (senility).  **Kidney**: two renal cysts in left kidney (7 cm in diameter).  **Lung**: tracheal edema, hyperinflated left lung, pulmonary edema and congestion.  **Skin**: interspecific interaction marks on fins.  **Spleen**: splenic congestion.  **Teeth**: exposed dental pulp. | **Cerebellum:** partially autolyzed, mild congestion. **Cerebral cortex:** mild congestion. **Cerebral meninges:** moderate congestion, mild multifocal to coalescent lymphoplasmacytic meningitis. **Colon:** mild to moderate diffuse lymphoplasmacytic colitis, associated to rare granulocytes. **Heart:** mild diffuse congestion, mild multifocal hemorrhage, minimum focal cardiomyolysis. **Intestine:** partially autolyzed. **Kidney:** moderate congestion, mild to moderate multifocal membranoproliferative glomerulonephritis, mild tubular degeneration, multifocal hyaline peritubular thickening. **Liver:** moderate congestion, mild ductal hyperplasia, hypertrophy of tunica media of hepatic arteries, mild to moderate macro e microgoticular degeneration, minimum lymphoplasmacytic periportal hepatitis. **Lung:** moderate to severe focally expansive bronchopneumonia fibrinosuppurative, associated to moderate congestion and multifocal hemorrhage, mild to moderate diffuse edema. **Lymph node:** expansion of the lymphoid follicles, associated to center follicular lymphocytolysis, mild to moderate diffuse granulocytic lymphadenitis, associated to edema and medullar and perifollicular distension. **Spleen:** marked expansion of perifollicular sheet, associated to lymphocytolysis, moderate to severe congestion, mild diffuse granulocytic splenitis, mild to moderate hemosiderosis.  **Adrenal** **gland:** partially autolyzed, NSFO. **Artery:** NSFO. **Skeletal muscle:** NSFO (artifacts). **Testicle:** partially autolyzed, hypospermia. |
| MM525 | Multiorgan congestion.  **Lung**: serosanguineous fluid in bronchi and thoracic cavity.  **Skin**: net entanglement marks in rostrum and head, and pectoral and caudal fins. | **Cerebellum** – partially autolyzed, NSFO. **Cerebrum** – partially autolyzed, NSFO. **Colon** – mild diffuse lymphoplasmacytic colitis, with presence of rare eosinophils. **Heart** – mild congestion. **Kidney** – mild peritubular congestion, mild edema and tubular degeneration. **Liver** – minimum lymphoplasmacytic periportal hepatitis, minimum multifocal lymphoplasmacytic mediozonal hepatitis, mild to moderate multifocal ductal hyperplasia, mild diffuse microgoticular degeneration. **Lung** – moderate to severe diffuse congestion, with presence of large quantity of alveolar macrophages desquamated full of hemosiderin, presence of granulocytes, and presence of bacterial colonies; mild multifocal hemorrhage; marked edema. **Lymph nodes, multiple, without defined localization:** mild diffuse granulocytic lymphadenitis, associated to lymphoid expansion and perifollicular edema. **Testicle:** physiologic azoospermia. **Thymus:** marked lymphoid expansion, presence of Hassall’s corpuscles, mild diffuse congestion. **Thyroid gland:** mild to moderate congestion. **Trachea:** peritracheal congestion. **Pancreas:** partially autolyzed, NSFO. **Lymph node, Peripancreatic:** marked follicular expansion, with lymphocytolysis centrofollicular, perifollicular edema with rare granulocytes. **Stomach: keratinized portion:** mild congestion; **glandular portion:** partially autolyzed, mild multifocal congestion.  **Adrenal gland:** NSFO. **Epididymis:** NSFO. **Skeletal muscle:** NSFO, presence of large amount of artifacts. **Skin:** NSFO. **Urinary bladder:** NSFO. |
| ii179266 | **Liver**: mild to moderate diffuse friable liver.  **Lung**: presence of multifocal encapsulated parasites, moderate to marked diffuse pulmonary congestion and edema,  **Uterus**: 62 cm-long fetus. | **Kidney:** congestion, partial autolysis. **Prescapular lymph node:** marked centro-germinal expansion, associated to mild centro-follicular lymphocytolysis. **Lung:** mild to moderate congestion, atelectasis (apparently an artifact). **Lymph node, mesenteric:** moderate multifocal to coalescent eosinophilic lymphadenitis, focal granulomatous lymphadenitis, with presence of giant cells. **Spleen:** mild to moderate acute eosinophilic splenitis, mild perivascular fibrinoid degeneration; mild to moderate periarteriolar lymphoid depletion. **Lymph node, without defined localization:** marked congestion and lymphoplasmacytic expansion.  **Adrenal gland:** advance autolysis. **Diaphragm:** NSFO. **Esophagus:** NSFO. **Heart:** NSFO. **Medulla:** NSFO, partial autolysis. **Placenta:** NSFO, partial autolysis. **Skin:** NSFO. **Stomach:** marked autolysis. **Urinary bladder:** NSFO. |
| ii137962 | Presence of umbilical cord, fetal folds and vibrissae. Meconium in the anal region. Hipostasis marks. Blood in the oral cavity. **Adrenal gland**: moderate to marked diffuse congestion.  **Subcutaneous tissue**: petechiae in the adipose tissue (head), especially in the periocular area and melon.  **Brain**: moderate to marked generalized central nervous system congestion.  **Kidney**: Moderate diffuse subcapsular renal hemorrhage and congestion.  **Lung**: moderate to marked multifocal diffuse pulmonary hemorrhage  **Heart**: mild to moderate multifocal petechiae in epicardium.  **Intestine**: congestion.  **Spleen**: moderate to marked diffuse splenic congestion and subcapsular hematoma.  **Stomach**: congestion. | **Adrenal gland:** mild corticomedullar congestion. **CNS fragments (cerebral cortex, cerebellum, medulla):** partial autolysis, NSFO. **Lymph node:** moderate acute lymphadenitis, with moderate lymphocytolysis and congestion. **Lymph node, mediastinal:** mild diffuse granulocytic lymphadenitis, moderate centrofollicular lymphocytolysis. **Lymph node, mesenteric:** mild granulocytic perifollicular lymphadenitis; moderate centro-germinal expansion, associated to moderate lymphocytolysis. **Heart:** minimum multifocal lymphoplasmacytic epicarditis, miocardium, NSFO. **Kidney:** partially autolyzed, mild congestion, presence of rare tubular cysts. **Liver:** mild to moderate ductal hyperplasia, mild to moderate multifocal periportal lymphoplasmacytic hepatitis, with rare presence of granulocytes, rare sinusoidal megakaryocytes. **Lung:** moderate multifocal hemorrhage; moderate diffuse congestion; mild acute granulocytic pneumonia, mild to moderate epithelial bronchiolar anisocytosis, with apparent formation of mild multifocal epithelial syncytia. **Pancreas:** NSFO, partial autolysis. **Peritoneum:** mild diffuse lymphoplasmacytic peritonitis. **Segment of reproductive tract:** mild to moderate multifocal hemorrhage. **Spleen:** moderate to marked congestion, associate to areas of hemorrhage; hyperplasia reticular, associated to lymphoid hypoplasia; presence of moderate quantity of megakaryocytes. **Stomach: keratinized portion:** moderate multifocal to coalescent perigastric hemorrhage; mild multifocal mononuclear gastritis. **Skeletal muscle:** mild multifocal coagulative rhabdomyolysis. **Thymus:** NSFO. **Thyroid gland:** moderate diffuse congestion, partial autolysis. **Urinary bladder:** mild to moderate congestion. **Small intestine:** NSFO, partial autolysis. |
| MM382 | Generalized congestion.  **Heart**: enlarged left ventricle, absent cardiac fat and blood cloth-filled cardiac chambers.  **Kidney**: subcapsular renal emphysema and mild cortico-medullar distinction.  **Left temporal nasal cavity:** profuse yellow discharge.  **Liver**: congestion, friable.  **Lung**: severe pulmonary congestion and edema.  **Ovary**: corpus luteum in the ipsilateral ovary (left).  **Pancreas**: congestion.  **Placenta**: clear abundant liquid and yellow opaque irregular spots.  **Skeletal muscle**: dark brown and friable musculature.  **Skin**: several cookie cutter shark (*Isistius* sp.) bite marks thorough the body, and scars consistent with intraspecific interactions.  **Small intestine**: edematous and thickened.  **Spleen**: congestion and presence of four accessory spleens (0.5-1 cm in diameter).  **Stomach**: hyperemic glandular mucosa with thick yellowish mucous attached to the villi, hyperemic pyloric stomach containing a foreign body (plastic bottle lid).  **Subcutaneous tissue**: presence of encysted *Monorygma grimaldii* parasites in the peritoneal perigenital region*.*  **Uterus:** fetus in an initial stage of development in the left uterine horn, metritis.  **Sinus**: purulent sinusitis.  The most likely cause of death was septic shock. The central nervous system was not examined. | **Cerebellum:** mild to moderate congestion. **Diaphragm –** NSFO. **Epididymis:** NSFO, partial autolysis. **Esophagus:** NSFO.  **Heart:** NSFO. **Kidney:** multifocal corticomedullar congestion; partial autolysis. **Liver:** moderate diffuse congestion; mild multifocal necrosis. **Lung:** moderate to marked diffuse congestion; mild diffuse lymphoplasmacytic bronchitis. **Lymph node, mediastinal:** lymphoid hyperplasia, with expansion of the germinal centers; centro-follicular lymphocytolysis; medullar expansion, with presence of moderate edema. **Pancreas:** mild congestion, moderate autolysis. **Prostate:** NSFO. **Skeletal muscle:** NSFO, partial autolysis. **Testicle:** NSFO.  **Small and large intestine:** marked autolysis. **Spleen:** marked congestion; lymphoid hypoplasia of marginal zone. |
| ii94246 | Moderate to marked generalized hemorrhage.  **Intestine**: mild intestinal parasitosis.  **Liver**: mild multifocal presence of cholesterol crystals.  **Skin**: sloughing, net entanglement marks in rostrum, caudal and dorsal fins, bite marks on the pectoral fin.  **Stomach**: mild to moderate multifocal gastritis. | **Lung:** moderate to severe congestion and edema, moderate autolysis. **Skeletal muscle:** NSFO, partial autolysis. **Liver:** mild mononuclear periportal hepatitis, with rare neutrophils; moderate diffuse congestion. **Kidney:** mild to moderate congestion; partial autolysis. **Prescapular lymph node:** moderate to severe lymphoid hyperplasia, with marked expansion of the germinal centers; moderate diffuse acute granulocytic lymphadenitis. **Mesenteric lymph node:** marked expansion of the germinal centers, mild to moderate granulocytic lymphadenitis, partial autolysis. **Glandular stomach:** marked autolysis; mild multifocal microabscesses. **Heart:** NSFO. **Liver:** marked autolysis. **Pancreas:** marked autolysis. **Cerebral cortex:** marked autolysis. |
